# *plinkQC*: an integrated tool for ancestry inference, sample selection, and quality control in population genetics

**DOI:** 10.1093/bioinformatics/btag612

**Published:** 2026-08-14

**Authors:** Maha Syed, Caroline Walter, Hannah V Meyer

**Affiliations:** Simons Center for Quantitative Biology, Cold Spring Harbor Laboratory, Cold Spring Harbor, NY 11724, United States; Simons Center for Quantitative Biology, Cold Spring Harbor Laboratory, Cold Spring Harbor, NY 11724, United States; Simons Center for Quantitative Biology, Cold Spring Harbor Laboratory, Cold Spring Harbor, NY 11724, United States

## Abstract

**Motivation:**

Population genetic analyses rely on high quality datasets that pass rigorous controls for sample and marker quality. Many analyses also require additional processing including identification of ancestry and sample relatedness. A software package that addresses all these common, yet crucial tasks is missing.

**Results:**

We have developed *plinkQC*, an R/CRAN package that combines these functionalities into a single software package with detailed vignettes for example applications. *plinkQC* determines the ancestry of study samples via a pre-trained random forest classifier that reaches 98% performance accuracy with just 5% of marker overlap between reference and user data. To obtain the maximal set of unrelated study samples, we developed a graph-based pruning method, taking both relationship estimates and sample quality into account. We demonstrate optimal sample selection on the 1000 Genomes project, where we retain an additional 71 samples compared to publicly available exclusion lists. Finally, *plinkQC* bundles these results together with per-individual and per-marker quality control checks into three simple functions and returns both the quality controlled dataset and quality control report about each step of the analysis.

**Availability and implementation:**

*plinkQC* is available as an R/CRAN package. The documentation and code are available on github: https://meyer-lab-cshl.github.io/plinkQC/ and https://github.com/meyer-lab-cshl/plinkQC_manuscript.

## 1 Introduction

Genetic association tests such as genome-wide association studies (GWAS) and quantitative trait loci (QTL) mapping are vital to our understanding of genetic risk factors and underlying biological mechanisms of physiological traits and diseases (Han *et al.* 2020, Schmiedel *et al.* 2022, Soskic *et al.* 2022). For robust and reproducible association results, rigorous quality control (QC) of the genotype data by removing low-quality samples and genetic markers is crucial. QC is commonly based on summary statistics of the genotypes, and is conducted on a genetic marker (most commonly single nucleotide polymorphism; SNP) and sample level (Anderson *et al.* 2010). Genetic marker QC includes removing markers with high missingness, low frequency and those not in Hardy–Weinberg equilibrium due to presumed genotyping errors. Sample-level quality controls include tests for sample swaps, often by checking if the predicted sex matches the assigned sex, the rate of missing markers, and the observed within-sample heterozygosity (Anderson *et al.* 2010). In addition, analyses often require controlling for a shared genetic background and/or direct relatedness within the study cohort. Depending on the purpose and assumptions of the downstream analysis, some or all of these filters may be applied to the data.

**Figure 1:**
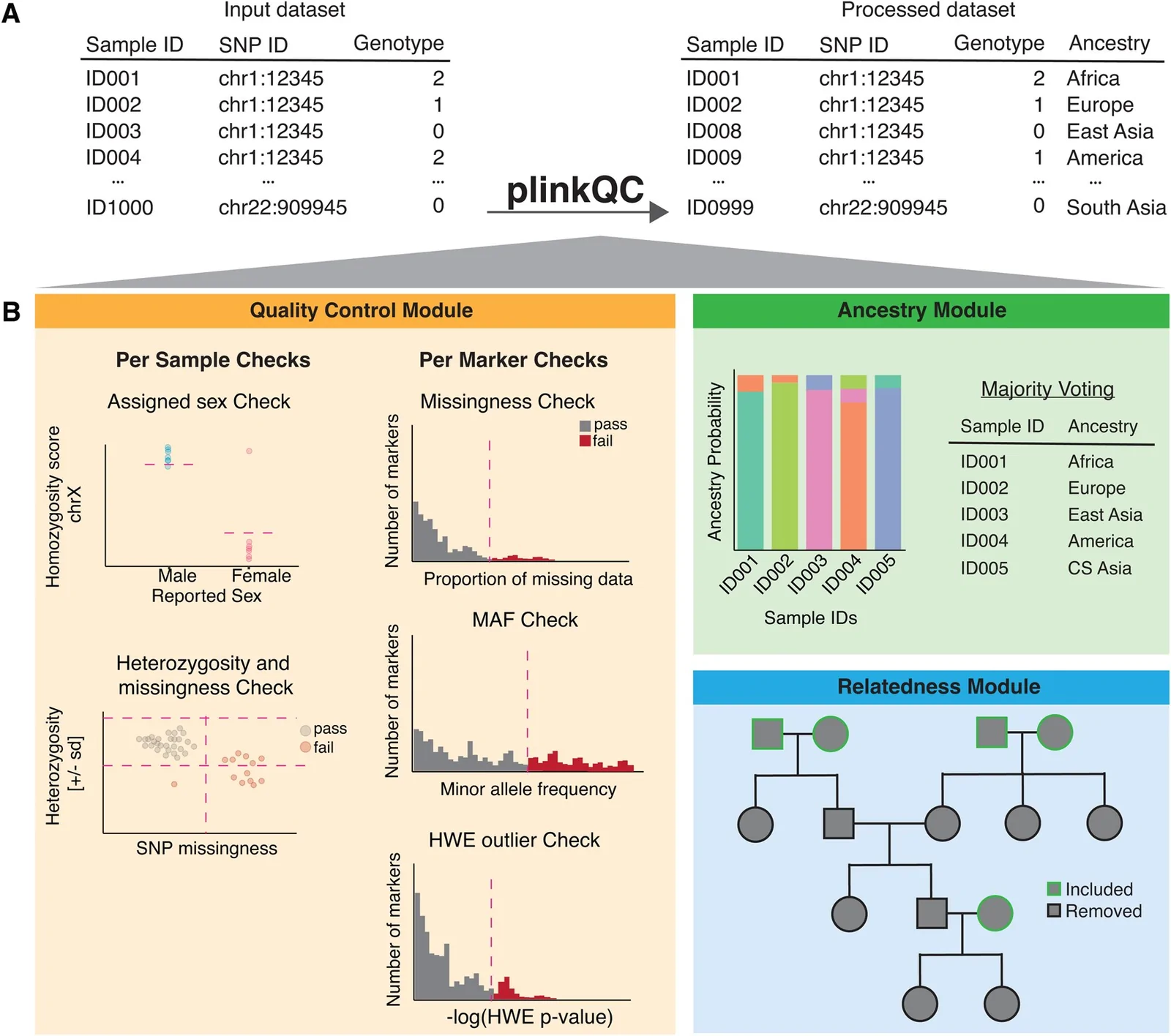
Standardized and modular genotype processing with *plinkQC*. (A) *plinkQC* takes in marker-based genotype data as input and returns the processed dataset based on user-specified modules and filters. (B) Quality control module: per-sample and per-marker checks for missing data, sample swaps and marker properties. Ancestry module: probabilistic and classification-based ancestry prediction. Relatedness module: relatedness identification and construction of maximal unrelated and high QC sample set.

Currently, rigorous genotype QC relies on the use of multiple tools to complete different tasks. For example, *King* (Manichaikul *et al.* 2010) is commonly used to remove related samples, while ADMIXTURE (Alexander *et al.* 2009) is used to determine the ancestry of samples. In addition, there is no tool that provides a standardized QC framework, with an accompanying report and direct output of the processed dataset with all filters applied. Here, we describe *plinkQC*, an efficient and all-in-one R/CRAN package to perform QC, ancestry identification, and selection of the maximally unrelated sample set (Fig. 1). It takes in variant-based genotyping data and returns a post-QC dataset of high-quality markers and samples, with predicted genomic ancestry and relatedness. For the latter, *plinkQC* implements (i) a classifier that predicts the genomic ancestry of human samples and (ii) graph-based relatedness pruning taking sample quality metrics into account. For QC, *plinkQC* computes underlying statistics with the commonly used command-line program *plink* and visualizes results in a QC report, which can be incorporated into larger analyses reports via the widely used *multiQC* tool (Ewels *et al.* 2016). The out-of-the-box genetic ancestry prediction is specific for human samples; for non-human studies, we provide vignettes for data processing and classifier training that can be incorporated into the provided functions. All remaining functionality is species agnostic. In summary, *plinkQC* bundles important functions to make genomic QC more efficient and accessible.

## 2 Workflow

*PlinkQC* takes in variant-based genotype calls in *plink* (either PLINK v1.9 or PLINK v2.0 format) or *vcf* format (visualized as human-readable tables in Fig. 1A). QC is conducted on a per-marker and per-sample level and users can choose from a set of QC filters (Fig. 1B). *PlinkQC* first computes and visualizes the QC statistics, providing the user with the choice of thresholding for subsequent application of marker and sample removal that fail QC.

**Per-marker QC:** The following three per-marker QC steps are recommended: (i) removal of genetic markers with high missingness rate as they might not have been genotyped properly; (ii) removal of genetic markers with a low minor allele frequency (or minor allele count; as specified by the user); they are often removed as downstream analyses would be underpowered; and (iii) removal of genetic markers with low *P*-value [high − log_10_(*P*-value)] in the test for Hardy–Weinberg equilibrium (HWE). This step is included as the strong deviation from HWE is assumed to reflect genotype calling errors. However, there could also be true signal captured in these markers and to avoid excluding biologically relevant markers under selection in case–control studies, often only the control samples are used to determine markers to remove.

**Per-sample markers:** The following three per-sample QC steps are recommended in each analysis: (i) prediction of the biological sex of each sample and comparison to the recorded sex to identify potential sample mislabeling or data entry errors; (ii) removal of samples with high missingness rate to identify poor quality samples; and (iii) removal of samples with outlying heterozygosity rate to flag samples that are potentially inbred or are contaminated with external DNA. In addition, downstream analyses might require assessing correlations between samples. Genomic data can be correlated between samples because of direct relatedness and/or broader population substructure. To support this, *plinkQC* includes tools for identifying related individuals and inferring genomic ancestry—key steps for controlling confounding effects and enabling robust downstream analysis. Users can choose which, if any, of these checks they would like to conduct.

## 3 Reference datasets

To develop the ancestry and relatedness modules, we relied on well-annotated human datasets, capturing a broad range of genetic backgrounds. The harmonized dataset of samples from the 1000 Genomes (The 1000 Genomes Project Consortium *et al.* 2015) and Human Genome Diversity Project (HGDP) (Bergström *et al.* 2020) served as an ideal reference dataset (Koenig *et al.* 2024), with 4151 individuals spanning seven continental ancestry groups.

In addition to representing a wide range of geographic and ancestral populations, the 1000 Genomes dataset also includes known familial relationships, providing a ground truth dataset for the development of our relatedness estimation module. After subsetting for samples from the 1000 Genomes project, the dataset contained 3202 samples. These samples were split into 2504 unrelated individuals and 698 individuals related to those in the prior dataset. From the 1000 Genomes pedigree file, there are 1799 samples labeled as having first degree relatives, and an additional 27 samples labeled as having second degree relatives. An overview of the data processing to develop the ancestry and relatedness modules are shown in  1, available as supplementary data at *Bioinformatics* online.

**Figure 2:**
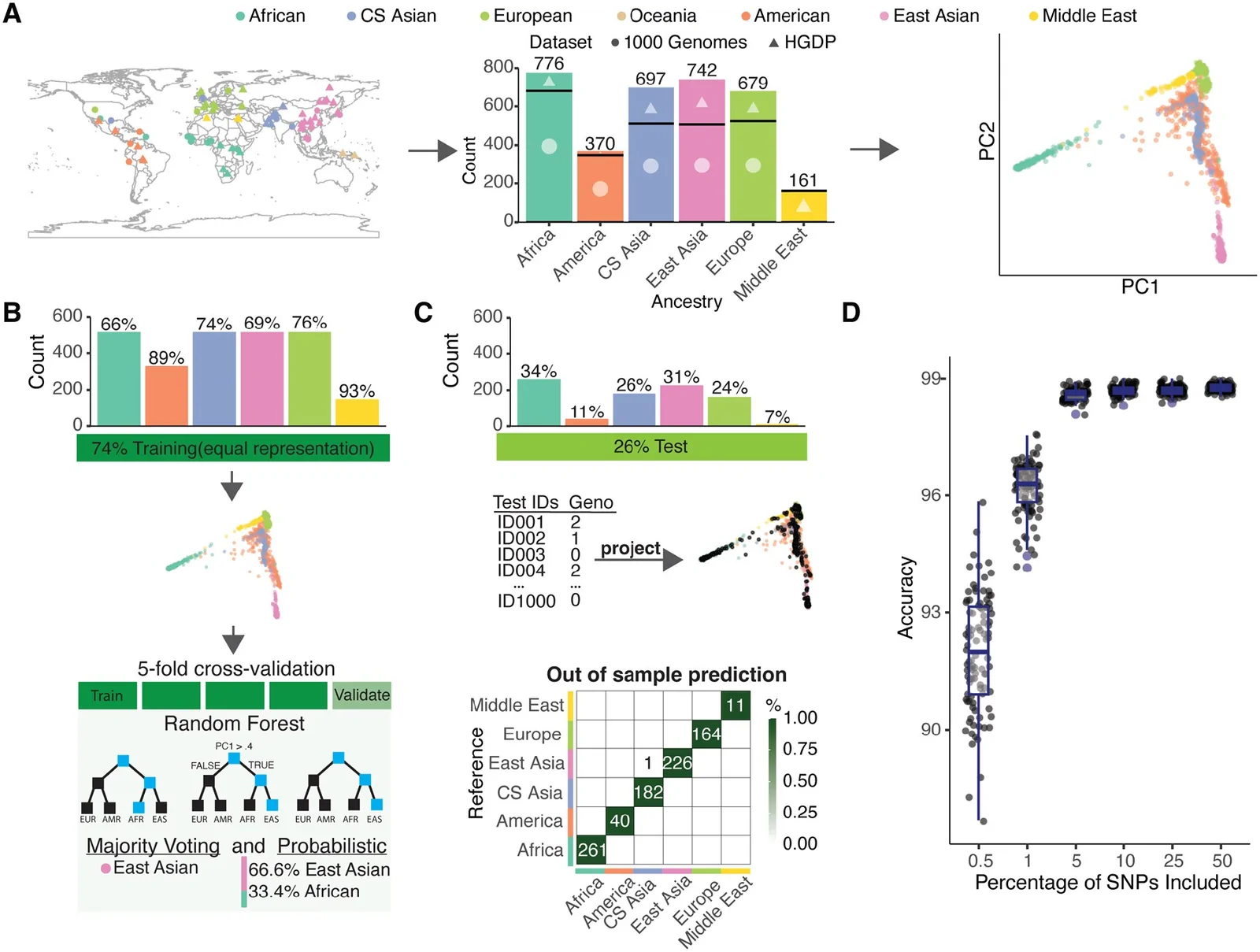
Ancestry identification with *plinkQC*. (A) Geographical distribution and sample counts of the full harmonized reference dataset consisting of 4150 labeled samples from the combined 1000 Genomes (3202 samples) and Human Genome Diversity Project datasets (948 samples). Principal components (PC) computed on the post-QC dataset of 3425 individuals with 229 020 independent SNPs (LD $r^{2}<0.2$). (B) Schematic of training data and random forest classifier taking PCs as features for ancestry prediction as either a probability vector or classification. (C) Ancestry classification of held-out test data (885 samples with 229 020 SNPs) by projecting test samples’ SNPs into PC embeddings based on training set. Classification accuracy is 99.9%. Absolute numbers for classification accuracy are indicated as values (zeros are not marked), colors indicate percentage (row normalized). (D) Accuracy of the algorithm on the held out test data with random downsampling of SNPs (*x*-axis; *n* = 100 random SNP sets).

For the development of the ancestry prediction module, we used all unrelated individuals of the combined 1000 Genomes and HGDP passing quality control. We combined the South Asia label from the 1000 Genomes dataset with the Central South Asia label from the HGDP dataset due to an overlap in the geographical regions. Additionally, we removed samples with Oceania ancestry due to small sample size (11 individuals). The final dataset contained 3425 samples across six continental ancestry groups: Africa, Central South Asia (CS Asia), Europe, admixed America, East Asian and Middle Eastern (Fig. 2A).

## 4 Ancestry identification

Population stratification can lead to spurious results in genetic association studies where associated variants are related to a group membership instead of the phenotype (Marchini *et al.* 2004). It occurs in data where subgroups display increased genetic correlation, which, in human data, is observed in individuals of common genomic ancestral backgrounds.

Without a sample pool that reflects the makeup of the population, the insights from genetic association studies are limited. Risk variants and effect sizes found in one ancestral group do not always correlate between ancestries (Carlson *et al.* 2013), which may be due to differences in linkage disequilibrium (LD) patterns (Sawyer *et al.* 2005) or varying pleiotropic effects of different genetic backgrounds. Additionally, ancestral groups may have different frequencies of risk alleles, and successfully identifying and including them in association studies enables novel risk allele identification (Cohen *et al.* 2005, Estrada *et al.* 2014). Thus, designing association tests that use all sample ancestries is critical to fully understand the effects of human genetic variation.

To account for population stratification in genetic association studies, many methods rely on principal components (PCs) of the sample genotypes. For cohorts composed of multiple ancestries, PCs can be used to define and divide cohorts into ancestry-specific subgroups for independent analyses and potential meta-analyses (Price *et al.* 2006, Peterson *et al.* 2019). Even within a cohort composed of one major continental ancestry, PCs may be used to exclude outliers (Momozawa *et al.* 2018) or capture small scale differences that can be accounted for as covariates in the model of choice (Cuomo *et al.* 2021).

Historically, the majority of human GWAS have been conducted on individuals from European descent (Mills and Rahal 2020). To reduce genetic heterogeneity, many of these studies removed non-European ancestral samples from their analysis (International Inflammatory Bowel Disease Genetics Consortium *et al.* 2017, Momozawa *et al.* 2018). However, with growing efforts to expand genomic data acquisition and genetic association studies beyond European ancestries, we need to be able to identify and retain multi-ancestral samples in the analysis (Nagai *et al.* 2017, Malaria Genomic Epidemiology Network *et al.* 2019, Thareja *et al.* 2021, Howley *et al.* 2025, Karczewski *et al.* 2025).

**Figure 3:**
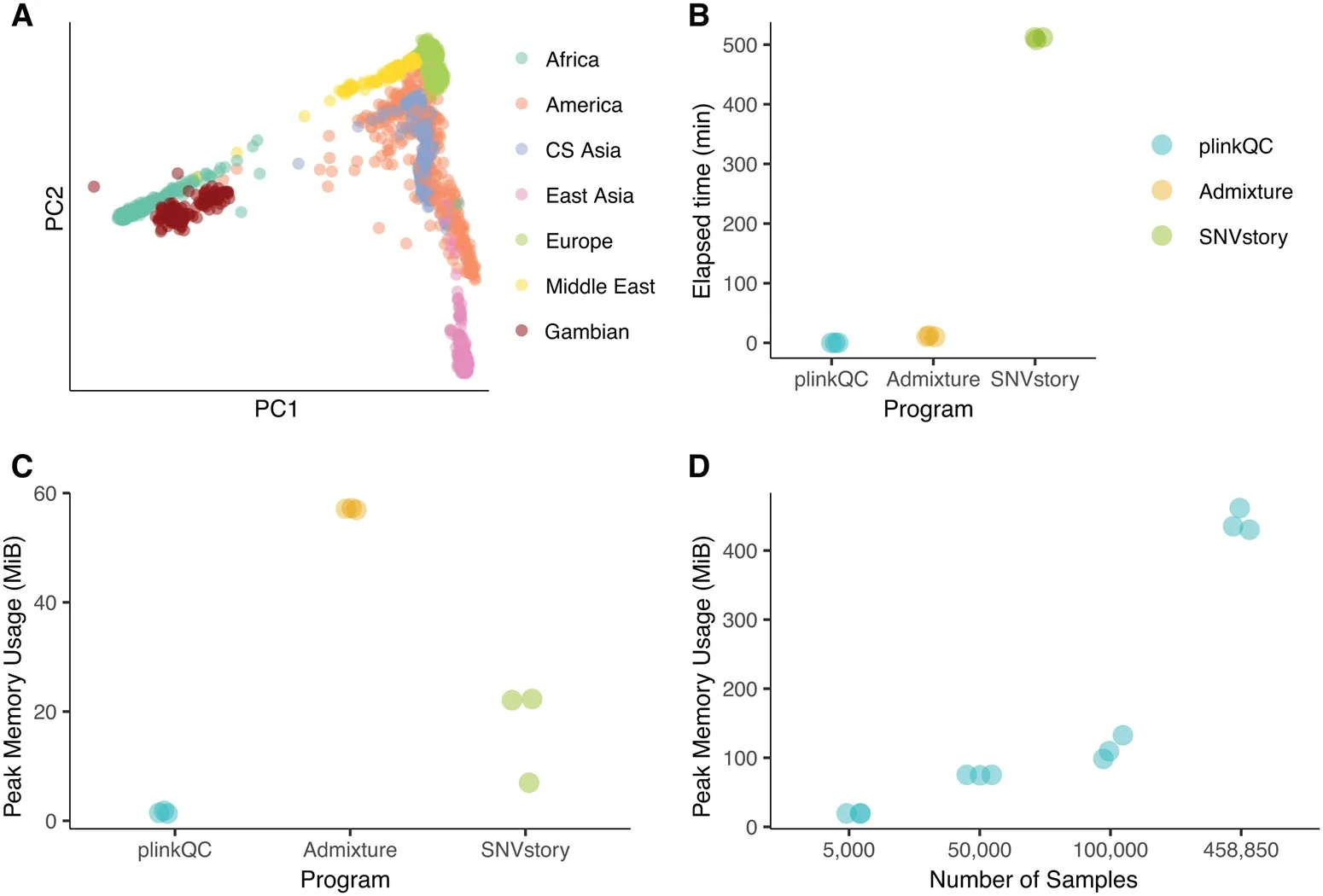
Validation and benchmarking. (A) Genotypes (14 095 SNPs) of 100 Fulani individuals from the Gambian Genome Variation Project projected into PC1 and PC2 of combined 1000 Genomes and HGDP reference panel. (B) Empirical runtime analyses for estimating ancestries of 100 Fulani individuals with *plinkQC*, ADMIXTURE, and *SNVstory*. The elapsed time to run ADMIXTURE is the combined time of unsupervised and supervised training and classification (Fig. 4, available as supplementary data at *Bioinformatics* online). (C) Peak memory usage for estimating ancestries of the 100 Fulani individuals with *plinkQC*, ADMIXTURE, and *SNVstory*. (D) Peak memory usage of *plinkQC* for estimating ancestries from simulated individuals generated with RESHAPE (Cavinato *et al*. 2024); *x*-axis represents subsamples of the full simulated dataset of 458 850 individuals. Panels B–D: individual points represent independent runs of the respective software on the same dataset.

Here, we trained a random forest classifier on the combined 1000 Genomes HGDP reference panel. It takes 11 projected sample PCs as features and returns estimated ancestry as either a probability vector or category (Fig. 2B). Our classifier achieves a 99.9% accuracy rate on the held-out test data, with one sample with East Asian ancestry misclassified as having Central South Asian ancestry (Fig. 2CTo evaluate the robustness of PC-based random forest classifier under conditions of incomplete genetic data, we simulated data with reduced marker overlap between user-provided input and the reference dataset. We first evaluated the robustness of the input (i.e. PCs) to missing data by calculating the Pearson correlation of PCs obtained from the full dataset versus PCs calculated from a subset of SNPs. Higher order PCs (PC 1–5) retain an average correlation above 0.9 even with just 5% of the SNPs shared between the reference and input (Fig. 3, available as supplementary data at *Bioinformatics* online). Using PCs calculated from just 5% of SNPs for classification yielded a mean prediction accuracy of 98.6%, close to the 99.9% accuracy with using the full set of markers (Fig. 2D.

**Figure 4:**
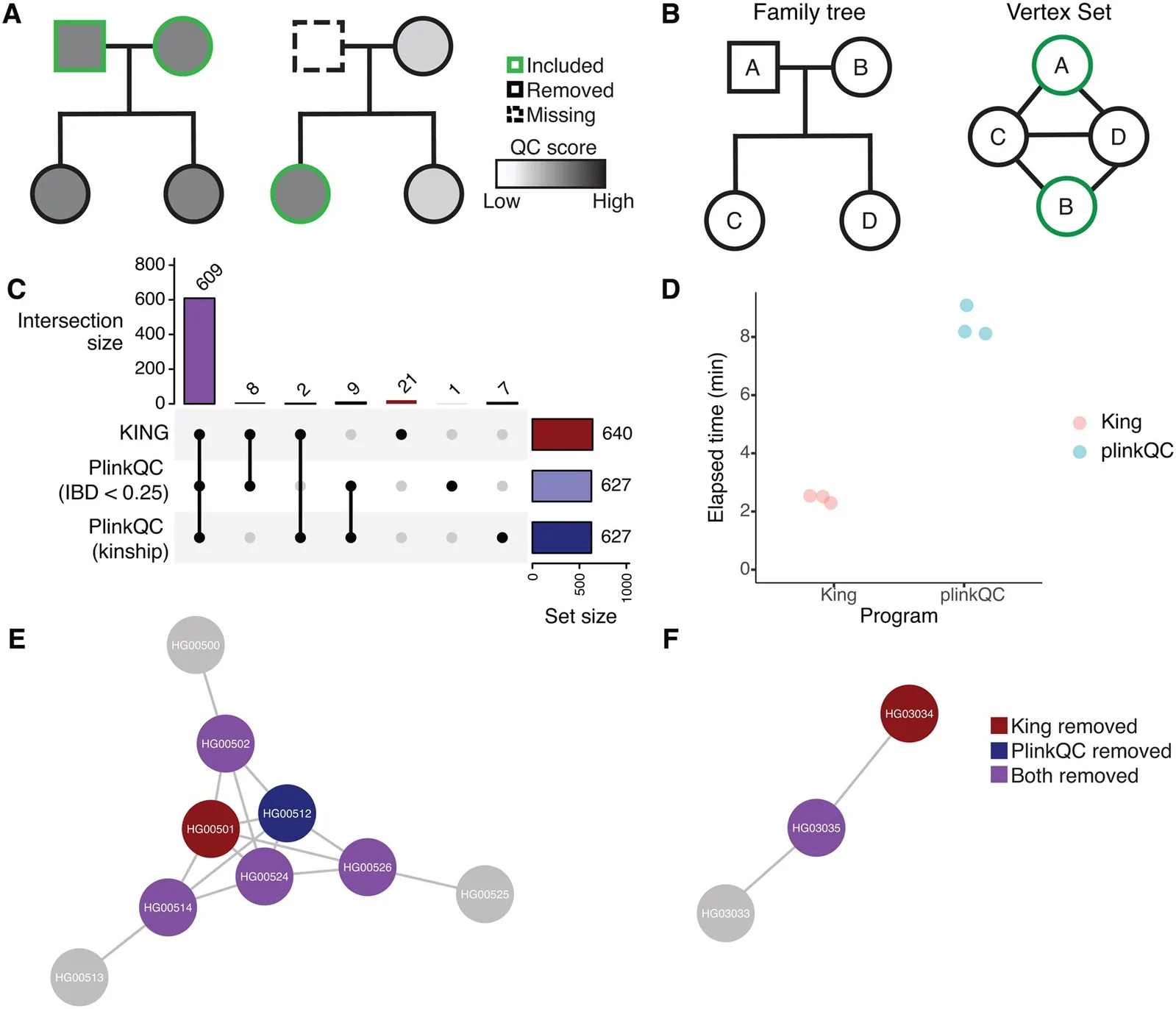
Relatedness identification and selection of unrelated individuals. (A) Schematic showing sample selection with pruning out related individuals based on retaining the maximum number of individuals with high QC scores. (B) Schematic of a four person family tree and its corresponding vertex set. (C) Samples from the 1000 Genomes dataset that are pruned out by *King*, *plinkQC* using the built in IBD scores, and *plinkQC* using *King* kinship scores as a relatedness matrix. The bars on the top show the intersection of samples that are removed and the bars on the right represent the total samples removed within each group. (D) User elapsed time taken to run *King* and *plinkQC* on the 1000 Genomes Dataset. Points are jittered for readability. (E) and (F) An independent vertex set of a family structure and trio found within the 1000 Genomes. A line between samples indicates a kinship score of above the *King* threshold for second degree relatives of 0.0884. Colors represent samples removed by *King*, *plinkQC* (kinship), or both.

As an external evaluation to assess how well our ancestry inference generalizes to a new dataset, we used SNP genotypes of 100 Fulani individuals from the Gambian Genome Variation Project (Malaria Genomic Epidemiology Network *et al.* 2019) (Fig. 3A). In addition to external validation, we also used this analysis to benchmark our ancestry inference method with two existing tools: ADMIXTURE (Alexander *et al.* 2009) and *SNVStory* (Bollas *et al.* 2024). ADMIXTURE can be run either in unsupervised mode, where the user simply specifies the number of expected populations or in supervised mode, where the user provides a merged reference and study dataset, effectively providing both number and labels of the expected populations. We set up ADMIXTURE in supervised mode in a two-step process. First, we ran it in unsupervised mode on the reference dataset and removed individuals with high levels of the admixture statistic; second, we ran it with population labels in supervised mode on this pruned dataset. We used the classifier built in supervised mode for predicting sample ancestry. As the reference dataset SNPs should match the study dataset, it is often necessary to retrain the model based on the study dataset. *SNVstory* uses a support vector machine directly on SNPs for ancestry identification. To set up *SNVstory*, we installed Docker Desktop and executed the software within the provided Docker Container. *plinkQC*, ADMIXTURE and *SNVstory* all predict 100% African ancestry. In runtime comparison, *SNVstory* takes >1000-fold longer (average runtime: 511 min) than *plinkQC* (<1 s) and ADMIXTURE (10.9 min; Fig. 3B). ADMIXTURE’s total time for the two-step training and projecting the 100 Fulani samples takes longer than *plinkQC*’s ancestry identification (time breakdown for each step given in Fig. 4, available as supplementary data at *Bioinformatics* online), which comes in addition to the upfront set-up time. Peak memory usage also varies by an order of magnitude, with average usage of 1.53 Mib for *plinkQC*, 17.1 Mib for *SNVstory*, and 57.1 Mib for ADMIXTURE (Fig. 3C). Importantly, *plinkQC*’s ancestry estimation does not rely on the full *N* sample by *S* SNP matrix (usually with S≫N) for inference, only requiring loading of the *N* sample by *P* number of PCs into memory. This is critical for analyzing genetic data from large-scale cohorts with 100 s of 1000 s of samples such as UK Biobank (Bycroft *et al.* 2018). To demonstrate this, we simulated genotypes across six ancestry groups for 458 850 individuals (corresponding to the size of UK Biobank) utilizing RESHAPE (Cavinato *et al.* 2024). Memory usage for ancestry classification with *plinkQC* did not exceed 500 MiB (average peak memory usage: 442.3 Mib; Fig. 3D) with a runtime maximum of <5 min (average runtime: 4.1 min). Classification accuracy was 100%.

For full flexibility, *plinkQC* also enables user-defined pre-trained classifiers in this module, extending the functionality beyond human studies or to different reference populations. To aid in the classifier design, we provide detailed vignettes for data processing and classifier training.

Overall, *plinkQC*’s ancestry identification module provides high accuracy and efficient out-of-the-box ancestry identification of large-scale, human genetics cohorts, with additional flexibility for custom analyses.

## 5 Relatedness

Genetic association analyses and heritability estimation may require removal of related individuals. However, the decision of which individuals to remove can influence the overall sample size. For instance, in a dataset containing two parents and one child, it may be more advantageous to retain both parents and exclude the child instead of retaining just the child. Within *plinkQC*, we developed a method to infer the largest independent vertex set of samples within a sample-relatedness graph to retain the maximum number of samples.

The approach begins by estimating identity-by-descent (IBD) between all pairs of samples. Predicted IBD is calculated by the proportions of the genome that is shared between two individuals (Milligan 2003, Purcell *et al.* 2007). If the pair of samples have a predicted IBD above a user-defined threshold, the samples are considered related. From the individuals labeled as related, we divide the sample pool into subgroups of related families, from which we construct family-specific vertex sets. Based on these, we calculate the maximum independent vertex set between individuals. If there are multiple options, for example one parent and two offspring, *plinkQC* chooses samples based on higher QC metrics.

To test the functionality of the relatedness pruning, we used the 1000 Genomes dataset, which contains 2504 unrelated individuals from the primary release and an additional 698 individuals related to the first sample set. The release of these two datasets was separated by time and the 2504 individuals labeled as unrelated do not necessarily build the maximal unrelated dataset out of the total of 3202 individuals. Here, we both address the question of the maximal unrelated set in the 1000 Genomes dataset and benchmark our approach to *King* (Manichaikul *et al*. 2010), a tool for detecting and filtering related individuals. In addition, since *plinkQC* and *King* use different metrics to estimate relationship, we also compared the *plinkQC* relatedness filter using *King* generated kinship scores as input (Fig. 4C).

The majority of samples removed by *King* and *plinkQC* (with either relatedness estimate) are shared (609 samples; Fig. 4C, intersection size). Looking closely at samples that are removed uniquely by any method, we see that they fall into two categories. They are either a pair of individuals, where removal of either individual reduces the relatedness in the sample set by same amount (e.g. removal of two out of three siblings; Fig. 4E) or *King* removes superfluous individuals e.g. two individuals from a trio when only one needs be removed (Fig. 4F). Overall, *plinkQC* with either IBD or *King*-based kinship as a relatedness estimate removes a consistently lower number of individuals than *King*. We used the 1000 Genomes pedigree file to determine how well these metrics match the annotated relatedness. We find that both algorithms pruned all family structures indicated as related. In addition, *King*- and IBD-based relatedness estimates uncovered related samples i.e. *King* kinship and IBD scores above the respective thresholds, that had no labeled relatives in the pedigree files that were subsequently removed in the relatedness filter. We also see four individuals labeled as secondary degree related which were not removed by *plinkQC* and three that were not removed by *King*. However, based on both IBD and kinship scores these individuals do not pass a threshold of secondary degree relationship (Table 1, available as supplementary data at *Bioinformatics* online). In summary, we identified that the maximal set of individuals without first and second degree relatedness in the 1000 Genomes dataset is 2575, increasing the sample size by 71 individuals.

## 6 Conclusion

We have introduced *plinkQC*, an open source R/CRAN package for genotype QC, relatedness, and ancestry estimation. Its QC module contains functions to conduct user-defined per-marker and per-sample quality control checks and returns both the cleaned dataset as well as an automated QC report with *multiQC* compatibility. Importantly, *plinkQC* offers additional functionality, with its relatedness and ancestry modules identifying maximally unrelated sample sets and ancestries of the study population. Combining these functions into single, software package with detailed vignettes for example applications, we provide an easy to install and to use solution for crucial, yet common tasks prior to genetic data analyses.

Custom random forest implementations have successfully been used for ancestry classification based on SNP genotypes (Koenig *et al.* 2024, Karczewski *et al.* 2025). Here, we trained a random forest classifier on a large human reference panel, which provides ancestry estimation in minutes even for biobank-scale data without study-specific retraining of the classifier. Further, we showed that classification accuracy remains excellent even if the overlap between SNPs in the study cohort and the training data is as little as 5%. *plinkQC*’s classification performance on external validation data is consistent with prior methods *SNVstory* and ADMIXTURE while improving both ease of setup and runtime. For ADMIXTURE’s unsupervised mode, it is common that multiple training runs using different initialization seeds have to be run to find a converging classifier. Additionally, users must include their own reference dataset, where reference and study dataset must contain the same SNPs. Thus each study dataset will likely require retraining of the reference dataset. In contrast, *SNVstory* contains a pre-trained classifier that can be used as-is on any new dataset and it offers more fine-scale ancestry resolution. However, multiple models are estimated for each sample, resulting in a significantly longer run time compared to ADMIXTURE’s supervised mode and *plinkQC*. Future improvements of the *plinkQC* ancestry module will include developing models for local ancestry estimation and training classifiers for ancestry estimation beyond continental ancestries. However, due to the small reference sample size for fine-grained populations in the current dataset, PCA projection of the external dataset will likely suffer from shrinkage to the mean, particularly for higher order PCs, so an additional correction method will likely be necessary (Privé *et al.* 2020, Wang *et al.* 2015). To allow for custom analyses of population stratification using different reference datasets or expanding the scope to non-human genetics, we provide detailed instructions in package vignettes for how to set-up, train, and implement new models. For instance, when additional samples from Oceania become available, the classifier can be easily adjusted to incorporate these.

Identifying the maximally unrelated sample set with *plinkQC* runs within minutes. While slightly longer than *King’*s runtime, *plinkQC* demonstrates larger sample retention compared to *King’*s identification process based on two estimates of relatedness.

Overall, *plinkQC* bundles together important genotype quality control functions as well as identifying related individuals and genomic ancestry identification while retaining efficiency and precision.

## 7 Material and m

### 7.1 Code and data availability

The package documentation and code are available at: https://meyer-lab-cshl.github.io/plinkQC/ and https://github.com/meyer-lab-cshl/plinkQC. Code for data analyses and manuscript figures are at: https://github.com/meyer-lab-cshl/plinkQC_manuscript. All analyses were conducted with R ≥4.0, *plink* version 1.90b6.21, and *plink2* version 2.0. 0-a.6.9LM. The user is required to install R and both plink versions. *plinkQC* internally handles usage of the required version with the ancestry module using *plink2* while all other functions use *plink*.

### 7.2 Datasets

#### 7.2.1 Combined 1000 genomes and human genome diversity project

We downloaded the vcf files for the autosomal chromosomes of the harmonized 1000 Genomes and HGDP datasets (Koenig *et al.* 2024), containing 189 381 961 variants from 4151 individuals. These variants composed of SNP, indels, and structural variants. For the purposes of ancestry identification and relatedness estimation, we merged the individual vcf files, converted the combined file to *plink* format and filtered for the 98 621 882 SNP variants only.

### 7.3 Relatedness estimation

#### 7.3.1 Data processing

From the merged dataset, we subset to samples from the 1000 Genomes project only. From these 3202 samples, we removed ambiguous SNPs (A/T and C/G mutations) and conducted plinkQC markerQC to remove SNPs with high missing rate (above 1% missing) or HWE-outliers (deviation significance threshold of 1e-5).

#### 7.3.2 Running *King* and *plinkQC*

We ran *King* on the processed dataset using king --unrelated--degree 2. The *King* kinship score matrix was calculated using king --kinship. For *plinkQC* based on IBD metric, we ran *plinkQC*’s check_relatedness using an IBD threshold of 0.25. For *plinkQC* based on *King* kinship, we ran relatednessFilter with a threshold of 0.0884 [second degree relative filter as defined in Manichaikul *et al.* (2010)] and supplying the *King* kinship matrix as input.

### 7.4 Ancestry estimation

#### 7.4.1 Data processing

To prune for related individuals, we first separated the dataset into the different reference ancestral populations. We then applied *plinkQC* per-marker checks to remove markers with high missing values (above 1%), rare variants (low minor allele frequency below 0.05), and a significance threshold of 1e − 05 for deviations from Hardy–Weinberg equilibrium. We removed related individuals within each ancestral label group using *plinkQC*’s check_relatedness function repeated the marker QC as above. We pruned for genetic markers in linkage disequilibrium (LD; $r^{2}<0.2$), with *plinkQC*’s pruning_ld function. We then used *plinkQC*’s per-sample checks to filter out samples with outlying heterozygosity (3 standard deviations above or below the mean) and high marker missingness (above 3%). Since there were only eleven Oceania samples left after sample quality control, we removed them.

#### 7.4.2 Random forest

From the 3425 samples with 229 020 SNPs that passed QC, we separated the data into 2540 samples for the training dataset and 885 samples for the testing component. We constructed the split such that the training data included an equal number of samples from different ancestral population, when possible, based on sample size (Fig. 2B, bar plot). We used plink2 --pca function to calculate the principal component analyses (PCA) eigenvectors and loading matrices for the training dataset. To have a consistent scale for projecting study data, we projected the training data on the loading matrices with the plink2 --score function. In the plink2 --score function, the genotype values are multiplied by the loading matrices and then divided by the total number of allele observations. While any missing or additional variants in the study data will not be included in the projection analysis, with missing data the number of allele observations decreases. The projected PC values were used as input data to train a random forest in *R* with the *randomForest v4.7.1.2* (Liaw and Wiener 2002) and *caret v7.0.1* (Kuhn 2008) library. For training, we used a grid search to optimize the number of PCs, trees, and variables sampled at each node of the random forest classifier (Fig. 2, available as supplementary data at *Bioinformatics* online). The parameters ranged from 1 to 200 trees, 2 to 20 PCs, and the number of variables sampled at node ranged from 2 to the number of PCs implemented. The out-of-bag error rate for the best performing model with 11 PCs and 25 trees is 0.47%. *plinkQC*’s ancestry identification program was run with the function superpop_classification.

#### 7.4.3 Simulating missing data

We simulated 100 random SNP subsamples from the held out test data to represent varying levels of missing data. To do so, we randomly selected a list of SNPs and extracted them into a new dataset. We used the extracted dataset as input for the superpop_classification function in *plinkQC* to determine the predicted ancestry and calculated the accuracy with the reference ancestry.

#### 7.4.4 Gambian data processing

FASTQ files were acquired for the Fula population from the Gambian Genome Variation Project. We used *bwa-mem2* (Vasimuddin *et al.* 2019) to align the reads to the hg39 reference genome, followed by sorting and merging the alignments for each sample with *samtools* (Li *et al.* 2009). Variant calling was done with GATK HaplotypeCaller and then GenotypeGVCFs to get vcf files. Then, we filtered low-quality markers with Quality of Depth (QD) < 2.0; Fisher Strand Bias (FS) > 60.0; RMS Mapping Quality (MQ) < 40.0; Mapping Quality Rank Sum Test (MQRankSum) < −12.5; and Rank Sum Test for Relative Positioning of REF versus ALT alleles (ReadPosRankSum) < −8.0 (McKenna *et al.* 2010). For *plinkQC* and ADMIXTURE, this vcf file was converted into plink2 format with parameters plink2 --vcf --not-chr X, Y, MT.

#### 7.4.5 *SNVstory*

*SNVstory* is released as a docker container that can be used with docker desktop (Bollas *et al.* 2024). We followed the instructions on the github (https://github.com/nch-cloud/snvstory) to install and ran *SNVstory* with parameters --genome-ver 38 --mode WGS --sample-pos all.

#### 7.4.6 ADMIXTURE

Admixture version 1.3.0 was installed using conda. ADMIXTURE requires corresponding SNPs in training and study population data. Thus, we filtered the reference training dataset of combined 1000 Genomes and HGDP samples (QC for this described above) for the SNPs that are shared with the Gambian dataset before pruning variants in $r^{2}<0.2$. We then trained ADMIXTURE in unsupervised mode (--unsupervised flag) for six random seeds and clusters were labeled manually. Visualizing the results, we observed that one trained model yielded inconsistent results, separating East Asian ancestry into two clusters and not differentiating between Middle Eastern and European ancestry, so we removed it (Fig. 5, available as supplementary data at *Bioinformatics* online). We averaged the predicted ancestry fractions across the remaining models and then selected ids >60% majority ancestry of their annotated ancestry and ran ADMIXTURE on this set in supervised mode. For final ancestry prediction, we projected the Gambian genotypes using the allele frequencies for each population learned from the reference population.

### 7.5 Runtime and memory usage comparison

Runtime comparisons for *plinkQC*, ADMIXTURE and *SNVstory* were run on a 64 bit AMD Ryzen 9 7900X 12-Core Processor Linux Ubuntu Workstation computer. Timing and memory usage runs were conducted three times with no other program running on the computer for consistency.

For performance evaluation of *plinkQC* with a biobank-scale cohort, we simulated 458 850 individuals using RESHAPE (Cavinato *et al.* 2024). We first split the testing subset of the harmonized 1000 genomes and HGDP dataset, excluding individuals of Middle Eastern ancestry (874 individuals; see section above for sample and data processing for this subset; Fig. 2C) into their ancestral groups. For each ancestry, we then used RESHAPE to simulate fifteen recombination events. We used the default HapMap genetic map as the required recombination map for RESHAPE. To create a sample size greater than the original reference populations, we repeated these simulations 525 times with different starting random seeds. Memory usage and runtime was measured on a Rocky Linux OS equipped with Intel Xeon Gold 6252 CPU with 2.10 GHz and 20 GB of requested memory. We randomly subsampled samples to get memory benchmarking on a range of sample sizes from 5000 to 458 850.

## Supplementary Material

btag612_Supplementary_Data

## Data Availability

The reference dataset underlying this article for the trained classifier is available in gnomAD at https://gnomad.broadinstitute.org/downloads#v3. The gnomAD v3.1.2 dataset was used. The dataset for the external validation is avaliable at the European Nucleotide Archive at https://www.ebi.ac.uk/ena/browser/home and can be accessed with project code PRJEB3013.
