# Supplementary material for: plinkQC: an integrated tool for ancestry inference, sample selection, and quality control in population genetics
Source: Bioinformatics. 2026 Aug 14;42(9):btag612. doi: 10.1093/bioinformatics/btag612 (PMC13533570; doi:10.1093/bioinformatics/btag612)
Supplement: Supplementary file 1 [file btag612_supplementary_data.pdf]

Supplement to: *plinkQC*: An Integrated Tool for Ancestry Inference, Sample Selection, and Quality Control in Population Genetics

Maha Syed [1], **Caroline Walter** [1], **Hannah V. Meyer** [1✉]

[1]Simons Center for Quantitative Biology, Cold Spring Harbor Laboratory, Cold Spring Harbor, NY 11724, USA

Supplementary Figures

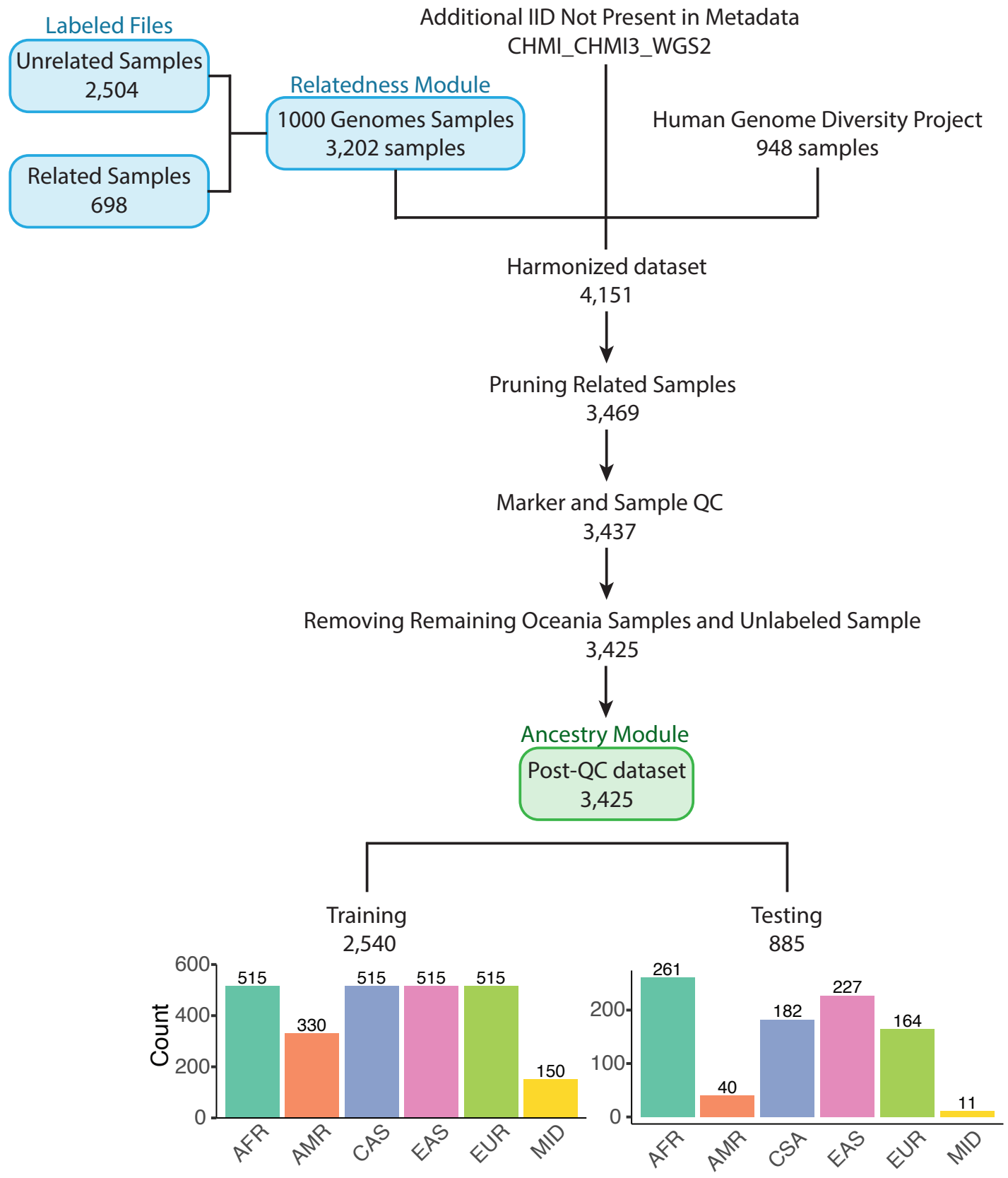

Supplementary Figure 1. Flowchart of the Reference Data. Reference data sets for developing and evaluating the relatedness and ancestry modules. Parameters for each step are described in detail in the Methods.

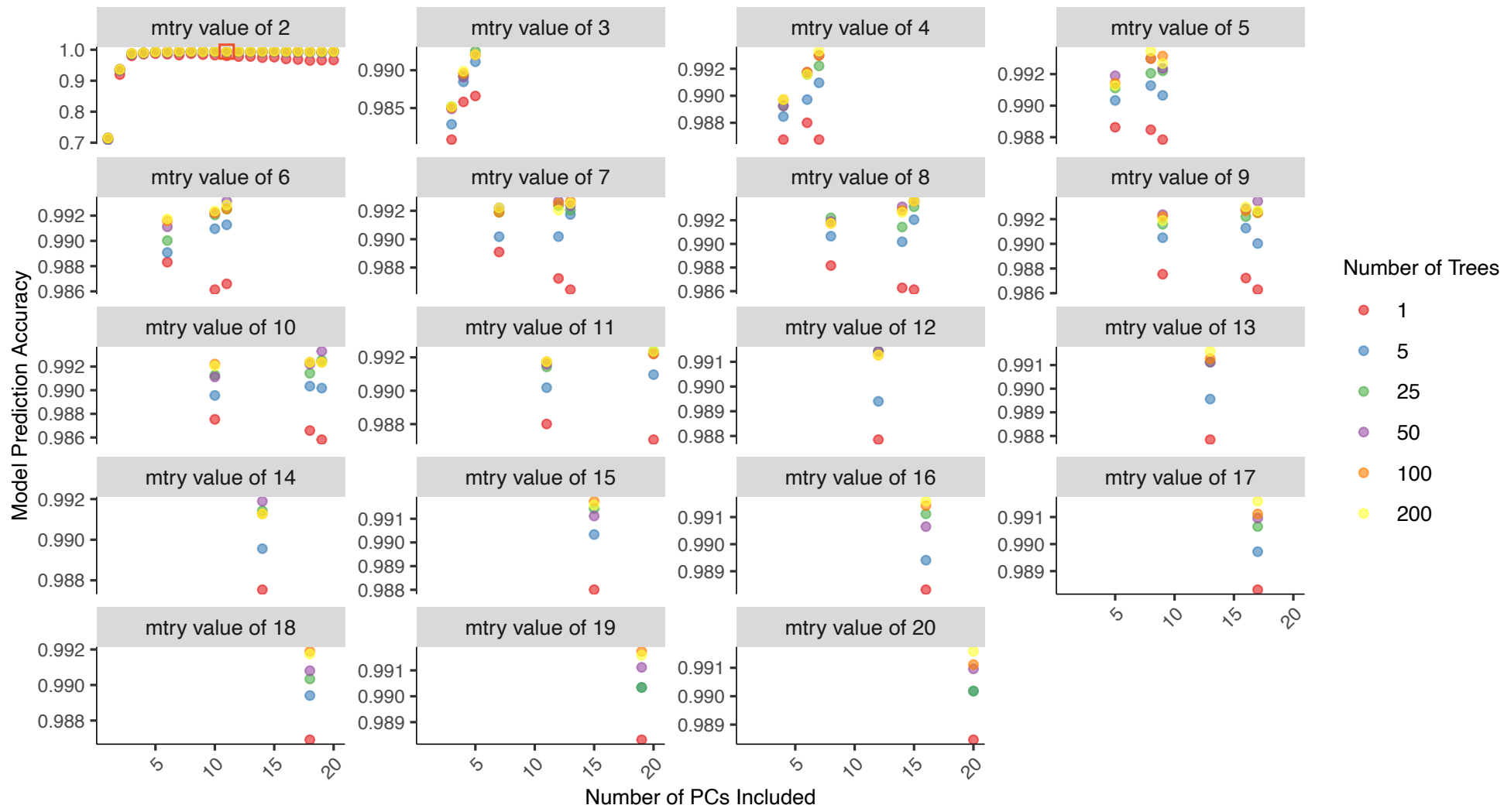

Supplementary Figure 2. Grid Search for Optimizing Parameters. Random forest parameter selection by grid search. The plot is faceted by mtry, the number of variables sampled at each node. The best performing parameters are 11 PCs, 25 trees, and 2 variables sampled at each node indicated by box.

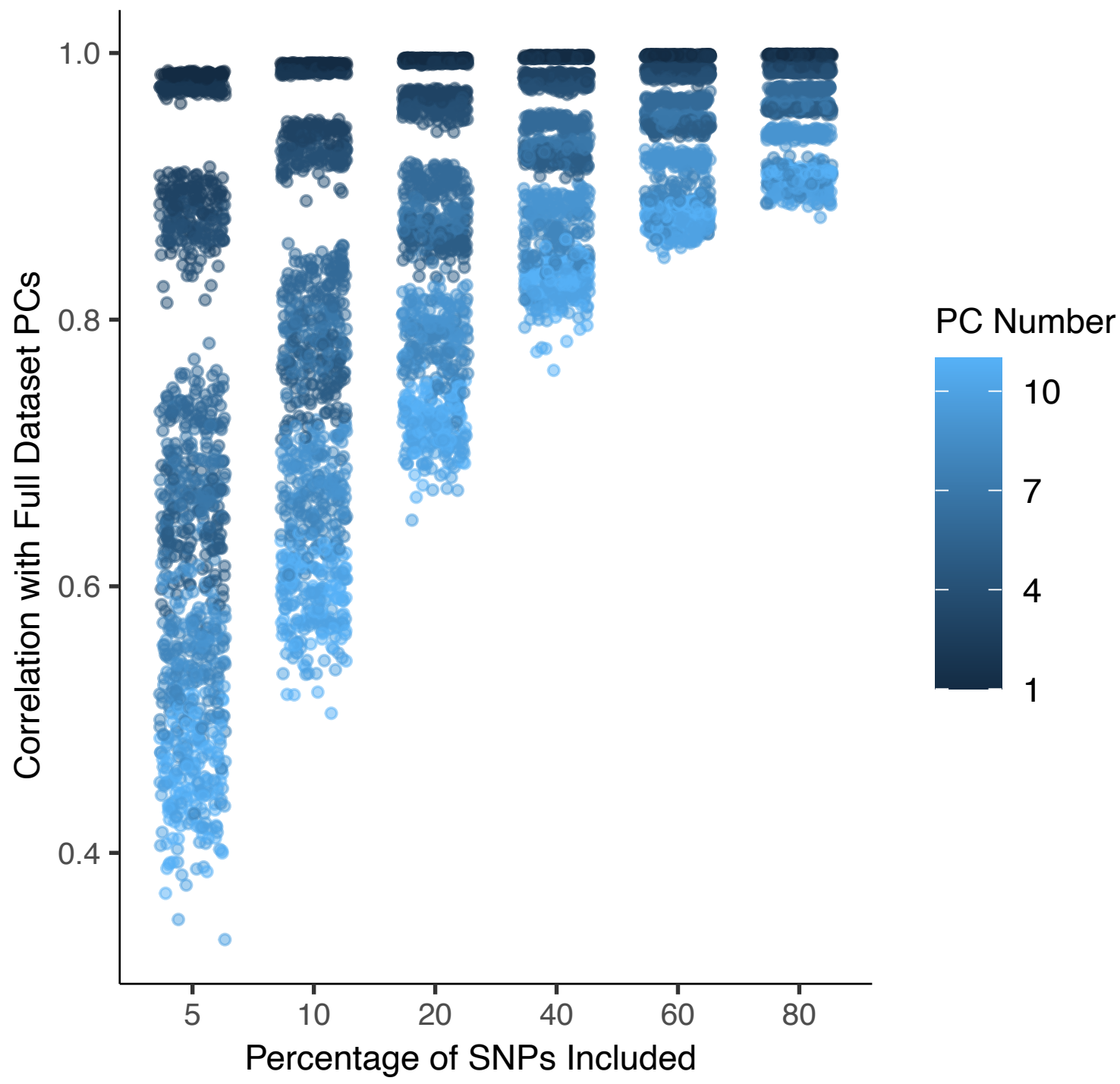

Supplementary Figure 3. Correlation of Projected PCs with Missing Data. Pearson correlation between PCs obtained from the ancestry test dataset with all SNPs (886 samples with 229,020 SNPs) and PCs calculated from a subset of SNPs (x-axis; n=100 random SNP sets). Each PC order is represented with a different color.

Supplementary Table 1. Kinship and IBD scores for pairs of individuals labeled as related in 1000 Genomes and not removed by *plinkQC* or *King*. Individuals with related individuals (labeled by 1000 Genomes) left in the dataset after pruning to only unrelated individuals with *plinkQC* or *King*. IDs italicized and underlined remain in the dataset with a labeled relative after pruning by *plinkQC* (IBD threshold of < 0.25), *plinkQC* (kinship threshold of < 0.0884), and *King*. IDs italicized but not underlined remain in the dataset with a labeled relative after pruning by *plinkQC* (IBD threshold of < 0.25) and *plinkQC* (kinship threshold of < 0.0884). The sample HG01983 has a kinship scores with another individual within the dataset above the threshold. Thus, as described in the main text, this is another example of King removing additional samples.

IID1	IID2	IBD	Kinship	Pedigree Label
NA12045	*NA07031*	0.0338	-0.0034	2nd order
NA19101	*NA19092*	0.1588	0.0624	2nd order
N19184	*NA19203*	0.0557	0.0044	NA19203 is labeled to have N19184 as a sibling; N19184 is not labeled to have NA19203 as a sibling
HG01936	*HG01983*	0.2378	0.0775	2nd order

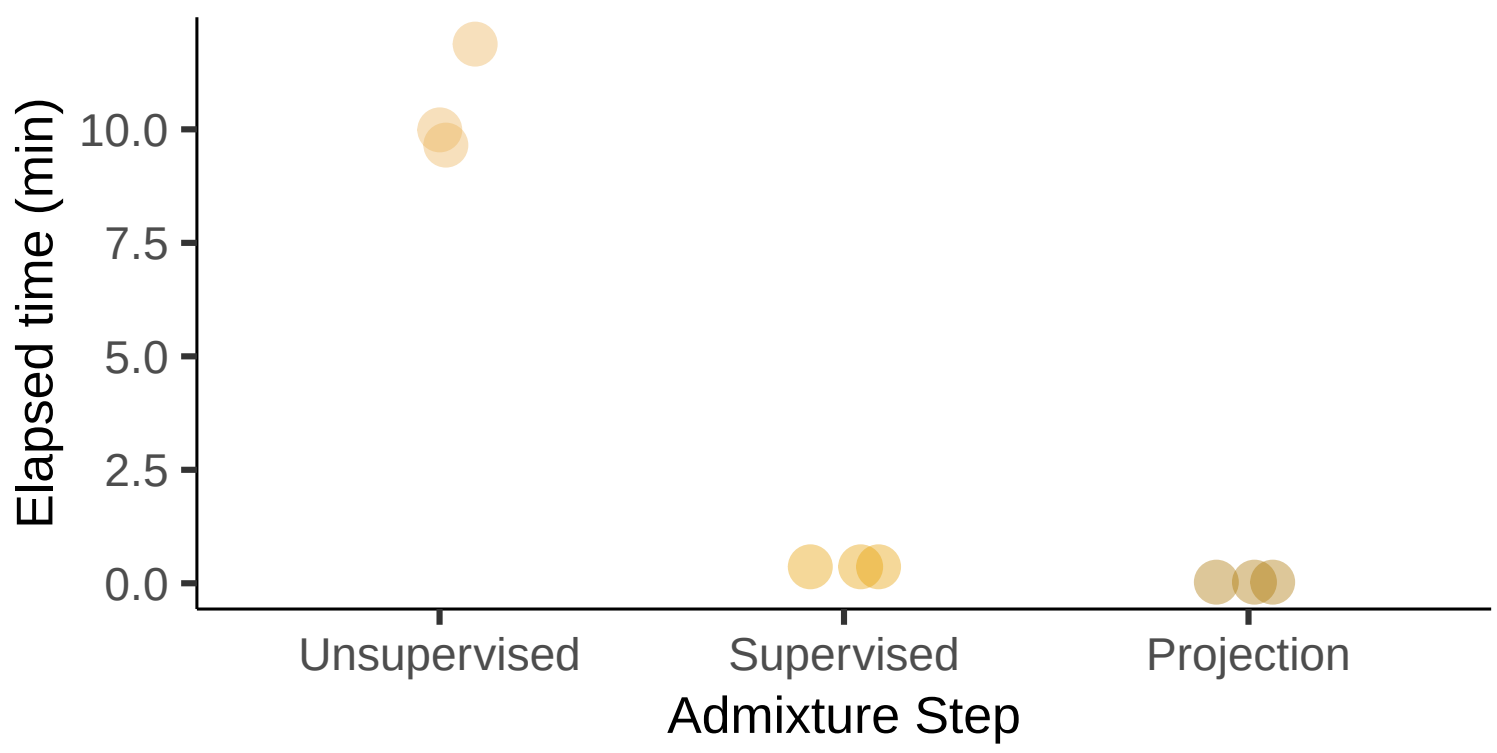

Supplementary Figure 4. Empirical runtime analyses for ADMIXTURE. Empirical runtime analyses for the three steps (unsupervised and supervised training and classification) needed by ADMIXTURE to estimate ancestries of 100 Fulani individuals from the Gambian Genome Variation. X-axis jitter added for readability.

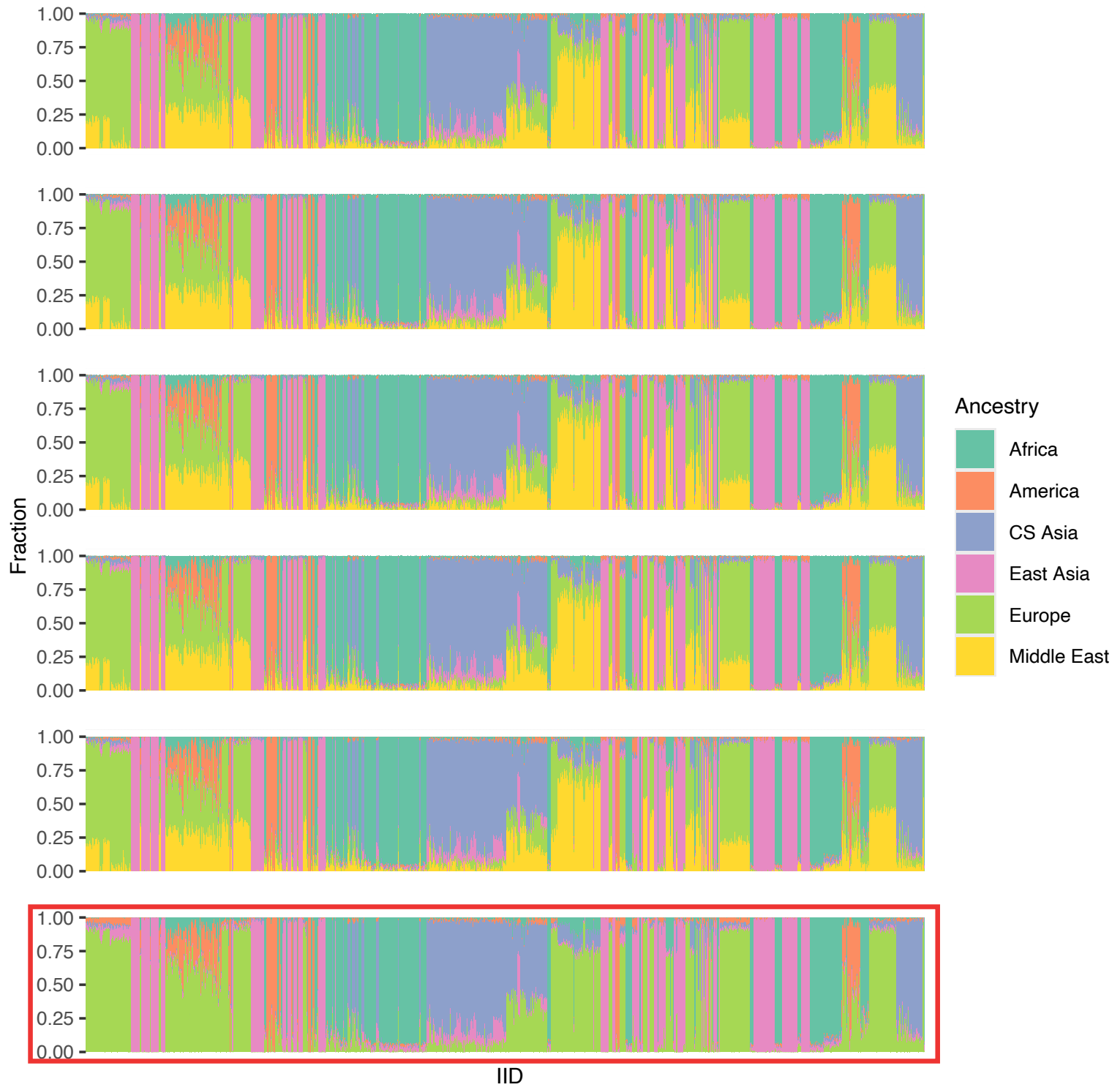

Supplementary Figure 5. ADMIXTURE classifier after unsupervised training with six random seeds. ADMIXTURE was run in unsupervised mode with six populations on the reference dataset for six different random seeds. The model indicated with a red rectangle was removed from downstream analyses for inconsistent results compared to the remaining five models.
